# Sulfated GAG mimetic peptide nanofibers enhance chondrogenic differentiation of mesenchymal stem cells in 3D *in vitro* models

**DOI:** 10.1093/rb/rbac084

**Published:** 2022-11-07

**Authors:** Seher Yaylaci, Mustafa O Guler, Ayse B Tekinay

**Affiliations:** Faculty of Medicine, Lokman Hekim University, Ankara 06800, Turkey; Pritzker School of Molecular Engineering, University of Chicago, Chicago, IL 60637, USA; Requalite GmbH, 82166 Gräfelfing, Germany

**Keywords:** peptide amphiphile nanofibers, *in vitro* chondrogenic differentiation, mesenchymal stem cells, 3D cell culture

## Abstract

Articular cartilage, which is exposed to continuous repetitive compressive stress, has limited self-healing capacity in the case of trauma. Thus, it is crucial to develop new treatment options for the effective regeneration of the cartilage tissue. Current cellular therapy treatment options are microfracture and autologous chondrocyte implantation; however, these treatments induce the formation of fibrous cartilage, which degenerates over time, rather than functional hyaline cartilage tissue. Tissue engineering studies using biodegradable scaffolds and autologous cells are vital for developing an effective long-term treatment option. 3D scaffolds composed of glycosaminoglycan-like peptide nanofibers are synthetic, bioactive, biocompatible, and biodegradable and trigger cell–cell interactions that enhance chondrogenic differentiation of cells without using any growth factors. We showed differentiation of mesenchymal stem cells into chondrocytes in both 2D and 3D culture, which produce a functional cartilage extracellular matrix, employing bioactive cues integrated into the peptide nanofiber scaffold without adding exogenous growth factors.

## Graphical Abstract

**Figure:**
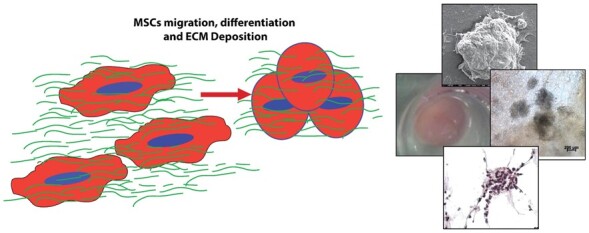

## Introduction

The current state-of-the-art treatment methods used for damaged cartilage tissues include bone marrow stimulation techniques such as arthroscopic debridement, microfracture, osteochondral autograft and allograft techniques [1]. Although these methods alleviate the symptoms in the short term and enable the recovery of low-scale mobility, in the long term, the newly formed tissue degenerates over time due to its mechanical weakness and this causes the surrounding healthy tissue to degenerate [2]. The problems associated with the long-term therapeutic effects of the currently used methods emphasize the need for alternative techniques [2, 3], which can restore the function of the cartilage tissue by providing biological signals with biocompatible materials [4]. 3D scaffold-matrix systems where the cells can attach, proliferate and differentiate have great potential for functional tissue replacement. When cartilage cells are removed from their 3D environment and transferred to a 2D environment, they can lose their chondrocyte characteristics and turn into fibroblasts. Therefore, 3D culture techniques are also needed to culture chondrocytes without phenotypic change. Different types of synthetic or natural materials can be used for this purpose [5] which can also transform mesenchymal stem cells into chondrocytes in 3D culture in the presence of various growth factors [6, 7]. In the 3D scaffold-matrix systems developed for cartilage tissue regeneration, the biochemical and biophysical properties of the native cartilage extracellular matrix, as well as its interaction with resident cells, should be addressed.

Articular cartilage mostly consists of hyaline cartilage tissue and provides a frictionless environment with the help of synovial fluid during the movement of two bones opposite the joint. Cartilage extracellular matrix, which is very dense in terms of collagen fibers and aggrecan molecules, constitutes the structural basis required to perform the mechanical function of the tissue. At the same time, glycosaminoglycan molecules give the tissue a net negative charge which allows retention of water that constitutes 70% of the tissue. The function of the cartilage tissue under compressive stress depends on the compression of this fluid and the collagen network structure that resists it [8]. Natural polymers such as collagen and hyaluronan can be blended to form 3D scaffolds that resemble the structure of cartilage tissue [9]; however, there are difficulties in obtaining natural polymers in a pure form. Harvested natural biopolymers may also contain harmful biological contamination, or might suffer from significant batch-to-batch variation that may occur during production processes [10]. On the other hand, synthetic polymers also have some disadvantages because of their low biocompatibility, biodegradability and the difficulties in simulating the extracellular matrix environment [1]. Synthetic peptide materials may provide an alternative to overcome the problems mentioned above [11]. Peptide amphiphiles are peptide-based synthetic materials that can self-assemble through hydrophobic interactions between the alkyl chains found at one end of the molecule and electrostatic interactions and hydrogen bonds between oppositely charged amino acids or through addition of charge-neutralizing chemicals [12]. In an aqueous environment, these fibers can form 3D hydrogels [13, 14]. By including biologically active amino acid sequences in the peptide amphiphile structure, the functional domains of the extracellular matrix that interact with the cells inducing them to proliferate or differentiate can be mimicked [15, 16]. For example, when the RGDS peptide sequence found in fibronectin was incorporated into the peptide amphiphile structure, the gels formed by these molecules provided a suitable culture medium for many cell lines to survive and grow [17]. The use of peptide-based hydrogels meets many important needs in cartilage tissue regeneration. First, the ability of hydrogels to encapsulate large amounts of water (by 99%) is important because of the creation of an environment similar to the aqueous structure of cartilage [18]. Peptide amphiphiles are biocompatible due to their peptide-based nature, injectable, which allows ease of use without invasive procedures, and can be metabolized in a reasonable period making way for the newly synthesized extracellular matrix without the need for surgical excision [19, 20]. Based on the extracellular matrix structure of the cartilage tissue, it is possible to develop a biochemically similar environment for healthy proliferation and differentiation of stem cells by using peptide amphiphiles. Mesenchymal stem cells have been shown to transform into cells expressing cartilage-specific molecules such as collagen II and aggrecan when cultured on peptide amphiphile molecules capable of binding TGF-β growth factor *in vitro*. In addition, when these molecules were injected into a damaged area created in rabbit joint cartilage, they could significantly repair the damage without the need for external growth factors [21]. However, the peptide amphiphile used in that particular study has low solubility and only interacts with TGF-β, while many different growth factors other than TGF-β take part during cartilage development such as IGF-1, FGF-2, BMP-2 and BMP-7 [1]. It is known that glycosaminoglycan chains located in the cartilage structure interact with more than one growth factor modulating their activity [22]. Thus, mimicking these natural glycosaminoglycans is an efficient way to capture growth factors released from resident cells to induce more regeneration responses in the damaged area. Previous studies that have utilized glycosaminoglycan mimetic (GAG-mimetic) peptide nanofibers have shown that such nanofibers support cartilage differentiation [23], bone differentiation [24] and wound healing [25]. These nanofibers have also been observed to enhance the transformation of chondroprogenitor cells into chondrocytes [23] and the transformation of rat mesenchymal stem cells into chondrocytes [26] in the 2D culture environment. However, a 3D culture that can induce differentiation of stem cells into chondrogenic fate has not been shown before. Since 2D cell culture is not suitable for chondrogenic cells, developing a 3D culture system that can induce chondrogenic differentiation of stem cells would be an invaluable tool for not only acclimatizing mesenchymal stem cells prior to transplantation into the damaged site for clinical applications but also for studying the molecular mechanisms of chondrogenic differentiation *in vitro*.

In this study, we aimed to synthesize and use peptide amphiphiles inspired by the glycosaminoglycan structure to bind to and increase the local concentration of different growth factors involved in the cartilage formation process and cartilage metabolism to enhance the regeneration of the damaged cartilage tissue. Here, we examined the role of the 3D gels formed by glycosaminoglycan-mimetic peptide amphiphile molecules in inducing mesenchymal stem cell differentiation into chondrocytes. We observed that these 3D nanofibrous gels supported the survival and differentiation of mesenchymal stem cells, mimicking the natural microenvironment of cartilage. Stem cells were cultured in 2D and 3D environments, and their responses in terms of viability and differentiation were compared. Unlike other studies using glycosaminoglycan-mimetic peptide materials, in this study, cells were cultured in the 3D environment to promote cell–cell interactions. Moreover, by increasing their exposure to naturally released growth factors, cartilage differentiation was induced without the use of exogeneous growth factors.

## Materials and methods

### Materials

9-Fluorenylmethoxycarbonyl (Fmoc) and tert-butoxycarbonyl (Boc) protected amino acids, Wang resin and 2-(1H-benzotriazol-1-yl)-1,1,3,3 tetramethyluronium hexaf luorophosphate (HBTU) for peptide amphiphile synthesis were purchased from NovaBiochem. Lauric acid and N,N-diisopropylethylamine (DIEA) were purchased from Merck. Other chemicals were purchased from Alfa Aesar or Sigma–Aldrich and used without any purification. Millipore Milli-Q deionized water was used during the experiments with a resistance of 18 MΩ cm mouse mesenchymal stem cell was purchased from Cell Biologics (C57-6043). All cell culture chemicals were purchased from Biological Industries (Kibbutz Beit Haemek, Israel) and cell culture consumables were purchased from Nest Scientific USA Inc.

### Synthesis of peptide amphiphile molecules

Three different peptide amphiphile molecules were synthesized using the solid phase peptide synthesis method. SO_3_-PA and K-PA were synthesized on Rink amide resin and E-PA was synthesized on Wang resin. All amino acids and resins had Fmoc protecting group. The amino acids were dissolved in dimethylformamide (DMF), O-(Benzotriazol-1-yl)-N, N, N′,N′-tetramethyluronium hexafluorophosphate (HBTU) and N, N-Diisopropylethylamine (DIEA) at a ratio of 2:1.95:3 and were added to the chain growing on the resin. The Fmoc protecting group of the amino acids added to the growing chain was cleaved by incubating the solid phase with 20% piperidine/DMF solution for 20 min before the next amino acid was added. The growing chain was incubated for 30 min in a 10% acetic acid/DMF solution to block the unreacted amine groups. After all the amino acids were added, lauric acid was added at the end of the chain in the same way. To separate the synthesized peptides from the resin, the solid phase was rinsed in a 95:2.5:2.5 solution of trifluoroacetic acid (TFA): triiopropylsilane: water for 2 h and the detached peptides were collected in a flask. The washing solution containing dichloromethane and TFA was removed by rotary evaporation and the peptides in the remaining solution were precipitated by adding diethyl ether and keeping at −20°C overnight. The peptide precipitate was collected by centrifugation and dissolved in water. After the peptide solution was frozen at −80°C, it was lyophilized and pulverized. The sequences, molecular weights and net charges of the peptide amphiphile molecules at pH 7 were as in Supplementary Table S1.

### Purification of synthesized peptide amphiphile molecules

The purity and molecular mass of the synthesized peptides were determined using the Agilent 6530-1200 QTOF LC-MS system with the addition of ESI-MS. Zorbax Extend-C18 2.1 mm×50 mm columns were used for basic conditions and Zorbax SB-C8 4.6 mm×100 mm columns for acidic conditions. As a gradient, 0.1% TFA/water and 0.1% TFA/acetonitrile solutions were used. Peptide amphiphile molecules were purified by reverse phase HPLC system. The columns used were Zorbax Extend-C18 2.1 mm×50 mm for basic conditions and Zorbax SB-C8 4.6 mm×100 mm for acidic conditions. As a gradient, 0.1% ammonium hydroxide/water and 0.1% ammonium hydroxide/acetonitrile were used.

### Visualization of synthesized peptide amphiphile nanofibers

Nanofiber formation was visualized by scanning electron microscopy. About 0.1% peptide solutions prepared in water were mixed on slides and the gels formed were passed through increasing concentrations of alcohol solutions (20%, 40%, 60%, 80% and 100%) for 10 min. The gels were then dried with a critical point dryer (Tousimis Autosamdri-815B). The dried gels were coated with 6 nm gold/palladium and images were taken under a scanning electron microscope (FEI Quanta 200 FEG SEM).

### Determination of the secondary structures by circular dichroism and zeta potential by Zetasizer

Circular dichroism (CD) measurements were taken using J-815 Jasco Spectrometer with the following parameters; 190–300 nm wavelength, 4 s digital integration, 1 nm band thickness and 0.1 nm data interval. Data was formed by taking the average of three consecutive measurements. Zeta potential measurements of individual PAs and self-assembled nanofibers were performed with a Malvern Zeta-ZS Zetasizer with a 5 × 10^−5^ M at the indicated volume ratios for each peptide.

### Coating of peptide nanofibers for 2D cell culture

Before starting the cell culture experiments, the cell culture plates were coated by mixing 1 mM peptide amphiphile solutions in a total volume of 150 μl/cm^2^ and the gels were allowed to dry overnight under the hood. The coated cell culture plates were then sterilized by keeping them under UV for 30 min. Supplementary Table S2 lists the peptides and mixing ratios employed in the coating process.

### Binding analysis of cells cultured on peptide amphiphile nanofibers

For cell binding experiments, cells were seeded at a density of 5×10^3^ cells/cm^2^ on peptide nanofiber-coated cell plates and bare tissue culture plates. For the cell binding analyses, cells were incubated in serum-free Dulbecco’s modified Eagle medium (DMEM) containing 50 μg/ml cycloheximide and 4% serum albumin for 1 h at standard cell conditions (37°C and 5% CO_2_) before starting the experiment. Since this medium prevents the cells from synthesizing new proteins, the effect of the extracellular matrix proteins that are normally synthesized by the cells on cellular attachment was eliminated. Cells bound on peptide nanofiber-coated surfaces were stained with Calcein AM (Thermo Fisher, C1430) for 1 and 3 h after cell seeding. Unbound cells were washed with phosphate buffered saline (PBS) just before staining. The images of the attached cells were taken under a fluorescent microscope (Zeiss Axio scope A1) and the number of cells in each photograph was counted using the Image J program [27]. Results normalized to Hour 1 TC*P* values.

### Spreading analysis of cells cultured on peptide amphiphile nanofibers

The spreading characteristics of the cells when cultured on the nanofiber scaffolds and tissue culture plates were examined by staining the actin fibers and taking fluorescent microscopy images at 1 and 3 h. Peptide coatings were prepared as mentioned above and the cells were seeded on nanofiber scaffolds and tissue culture plates in maintenance medium (Supplementary Table S3). At 1 and 3 h, cells were fixed for 10 min in 4% paraformaldehyde/PBS. Cell membranes were then punctured with 0.1% Triton X-100/PBS to allow the dyes to penetrate into the cell. F-actin fibers were stained with TRITC conjugated phalloidin dye (Merck, P1951) and cell nuclei were stained with TO-PRO-3 dye (Thermo Fisher, T3605). The samples were examined under a confocal microscope (ZEISS LSM 510).

### Viability analysis of cells cultured on peptide amphiphile nanofibers

Cells were seeded on peptide nanofiber coated plates and bare tissue culture plates in maintenance medium at a density of 5×10^3^/cm^2^. Four wells were used for each group and the cells were stained at 24, 48 and at 72 h of culture with Calcein AM (Thermo Fisher, C1430) and visualized by a (Zeiss Axioscope A1) fluorescence microscope. At least six images were taken from each well and the stained cells per image were counted using the Image J program [27]. Results normalized to Day 1 TC*P* values.

### Differentiation analysis of cells cultured on peptide amphiphile nanofibers

Before the cells were seeded on cell culture plates, the wells were coated with 1 mM peptide amphiphile solutions in a total volume of 300 μl and the gels were allowed to dry overnight under a laminar flow hood. The coated cell culture plates were then sterilized by keeping them under UV for 30 min. In order to promote cell differentiation, cells were seeded at a density of 5×10^4^ cells/cm^2^ and cultured in various mediums including differentiation-inducing media (Supplementary Table S3).

#### Monitoring of morphology of differentiating cells cultured on peptide nanofiber nanofibers

Differentiating cells were visualized on Day 7 by optical microscopy (Zeiss Axio A1) and scanning electron microscopy. For scanning electron microscopy, cells were seeded on peptide nanofiber coatings prepared on 15 mm diameter cover-slips in 24-well cell culture plates with different medium compositions shown in Supplementary Table S3. After discarding the medium on Day 7, samples were washed with PBS and the cells were fixed first with 2% glutaraldehyde and then 4% osmium tetroxide at room temperature for 1 h each. After the fixation process, the cells were washed with PBS and dehydrated. For dehydration, cells were incubated for 10 min at increasing alcohol concentrations (100%, 95%, 80% and 70%) each. The samples were dried in a critical point dryer (Tousimis Autosamdri-815B) to protect the structure and remove the ethanol. Samples on glass were coated with 6 nm Au/Pd before imaging with a scanning electron microscope (FEI Quanta 200 FEG SEM).

#### Analyses of cartilage-specific protein synthesis of cells cultured on peptide amphiphile nanofibers

Mouse mesenchymal stem cells cultured on peptide nanofibers were stained with cartilage-specific anti-Collagen-II antibodies to characterize cartilage differentiation. After coating the cover slips with peptide nanofibers as mentioned above, the cells were seeded at a density of 5×10^4^ cells/cm^2^ in parallel plates for Days 7 and 14. On the specified days, cells were washed with PBS after medium was discarded. Then, the cells were fixed for 15 min with 4% paraformaldehyde and treated with 0.1% Triton-X/PBS for 15 min to puncture holes in the cell membrane. The cells were then blocked for 30 min in 10% serum albumin to prevent nonspecific binding. After blocking, cells were incubated with anti-Collagen-II antibody (Abcam, ab34712) at a concentration of 2 μg/ml for 1 h at room temperature on a shaker. After the primary antibody solutions were removed, the cells were washed 3 times with PBS for 5 min and incubated with the rabbit-specific secondary Cy3-conjugated antibody (Merck, AP124C) at a dilution of 1:200 for 1 h in the dark on the shaker. After incubation, cells were again washed 3 times with PBS for 5 min and incubated with DAPI (Millipore, 508741) for 20 min for nuclear staining. After staining, cells were washed and coverslips were fixed on slides. The samples were examined under a confocal microscope (ZEISS LSM 510).

#### Gene expression analysis of cells cultured on peptide nanofiber nanofibers

In order to examine the differentiation of mouse mesenchymal stem cells on peptide nanofibers, expression levels of cartilage specific genes (Collagen II, Aggrecan) were examined by qRT–PCR method. After coating the cell culture plates with peptide nanofibers as mentioned above, the cells were seeded in parallel cell culture plates at a density of 5×10^4^ cells/cm^2^ for 7 and 14 days in different medium compositions shown in Supplementary Table S3. For control group, cells were cultured on uncoated bare tissue culture plate in maintenance medium were used. On the 7th and 14th days of culture, cells were detached from the cell culture plate with 1 ml/cm^2^ of Trizol and transferred to Eppendorf tubes. Samples were centrifuged at 15 000 rpm for 17 min at 4°C, and the upper transparent phase was transferred to new Eppendorf tubes and treated with isopropanol. After waiting for 10 min at room temperature, the samples were centrifuged at 15 000 rpm for 12 min at 4°C. The RNA pellets were washed by adding 1 ml of 70% ethanol to the tubes and centrifuging at 15 000 rpm for 8 min at 4°C. This process was repeated twice. After washing, the pellets were left in a laminar flow cabinet for 20 min to evaporate ethanol and dissolved in 30 µl of distilled water, after which their concentrations were measured using Nanodrop 2000 spectrophotometer (Thermo Scientific). RNA samples were stored at −80°C until qRT–PCR analyses. cDNA synthesis and qRT–PCR reaction from RNA samples were performed using ‘Super Script III Platinum SYBR Green One-Step qRT–PCR Kit’ (Invitrogen). The reaction conditions were as follows; 5 min 55°C, 5 min 95°C, 40 times 15 s 95°C, 30 s 60°C and 1 min 40°C, followed by melting curve analysis. The Ct values obtained were normalized according to the comparative CT method with respect to the expression of GAPDH gene [28].

### Differentiation analysis of cells cultured in 3D peptide nanofiber hydrogels

3D cell cultures were performed for the E-PA/K-PA and SO_3_-PA/K-PA hydrogel formulations. The PA hydrogels’ viscoelastic properties were examined using an Anton Paar Physica RM301 Rheometer with a 25-mm parallel plate design. The aforementioned ratio of 10 mM PA solutions was combined to generate a neutral hydrogel system. Analyses of rheology were conducted in both water and HEPES buffer. The gap distance was 0.5 mm, the angular frequency was 10 rad/s and the shear strain was 0.1%. All tests involving time sweeps were conducted at room temperature. Three independent measurements were carried out. In order to form 100 μl hydrogels, E-PA, K-PA and SO_3_-PA PA solutions at 10 nM were prepared in HEPES buffer solutions. In cell culture wells, 10 nM PA solutions were mixed with cell solution prepared in K-PA. In this study, we attempted to induce chondrogenic differentiation using micromass culture. Micromass culture, which was initially used to explore endochondral skeletal development in chicken embryos, provides a 3D environment for cell–cell interactions similar to those observed during embryogenesis. When researchers compared the potential of MSCs from bone marrow (BM) to induce chondrogenesis in a micromass system vs a pellet system, they concluded that the micromass system was superior [29]. We utilized 5×10^6^ cells per 100 μl of hydrogel and determined the cellular density according to the acceptable range in the literature. In order to equally distribute cells within the hydrogel and maintain its integrity, the cell solution produced in K-PA was injected into either E-PA or SO_3_-PA. The ultimate cell count in each hydrogel was 5×10^6^ cells. After forming 3D cell cultures, the cells were incubated for 14 days in maintenance medium and the medium was changed every two days. The composition of maintenance medium is defined in Supplementary Table S3.

#### Monitoring of cartilage-specific glycosaminoglycan and collagen synthesis of cells cultured in 3D peptide gels

At the end of Day 14, hydrogels were fixed in 4% paraformaldehyde for 48 h at 4°C and then dehydrated in a graded series of ethanol and cleared in two changes of xylene. Samples were embedded in paraffin blocks and sectioned at 5 µm thickness by microtome (Leica RM2125 RTS). For glycosaminoglycan imaging, sections were stained with Safranin-O (Merck, S8884) for 5 min and Fast Green (Merck, F7258) for 5 min and for hematoxylin and eosin (H&E) staining sections were stained with Mayer’s Hematoxylin (Merck, H9627) for 5 min and Eosin (Merck, 230251) for 45 s. Then, sections were dehydrated in graded ethanol solutions and cleared in xylene. Slides were mounted by Histomount^®^ mounting medium (Thermo Fisher, 008030) and imaged by light microscopy (Zeiss, Axio Scope). For immunohistochemical staining, slides were treated with an antigen retriever (Sigma, C9999) to uncover epitopes for 15 min at 37°C after rehydration steps. After blocking for 2 h at room temperature, sections were incubated with primary Collagen II (Abcam, ab34712) and Sox-9 (Abcam, ab185230) antibodies at 4°C overnight. Sections were then washed extensively with TRIS-buffered saline (TBS, 10×), with Triton™ X-100 (0.01% vol/vol) and treated with secondary antibody (Merck, AP124C) at a dilution of 1:500 for 1 h at room temperature to detect bound primary antibodies. Then, nuclei were stained with DAPI (Millipore, 508741). After staining, cells were washed and coverslips were fixed on slides. The samples were examined under a confocal microscope (ZEISS LSM 510).

### Statistical/data analysis

The mean and standard error (SEM) was calculated for all variables. Normality test was used to determine whether the distribution of all experimental group variables was consistent with a Gaussian distribution. Differences between cell groups were tested with the non-parametric Wilcoxon Matched-Pairs Signed Ranks Test. In all analyses, the difference was considered statistically significant if the associated *P* values are less than 0.05. Calculations were made with GraphPad Prism (Version 9; Graph Pad Software, San Diego, CA, USA).

## Results and discussion

### Peptide amphiphile molecules self-assemble into nanofibrous structures when mixed

**Figure 1.**
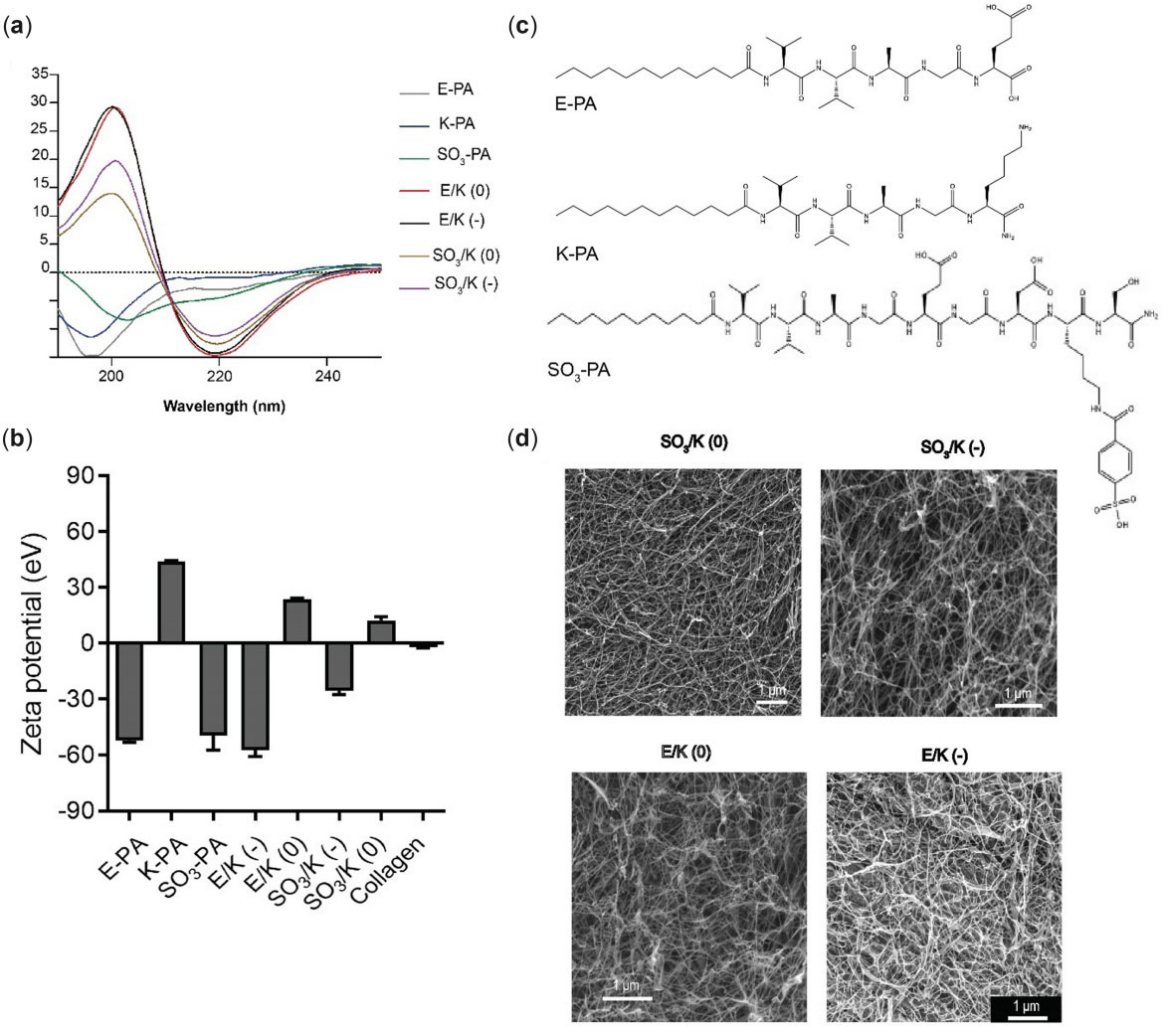
(**a**) Circular dichroism spectra of single peptide amphiphile molecules and nanofiber gels. (**b**) Zeta potentials of individual PAs and self-assembled nanofibers. (**c**) Chemical structures of PA molecules. (**d**) Scanning electron microscope micrograph of indicated nanofibers. Values represent mean±SEM, *n* = 3 (***P < 0.0001, **P < 0.01, *P < 0.05).

Peptide amphiphiles are peptide molecules with an alkyl chain at one end. These molecules can be easily designed to be soluble by including charged amino acids in the peptide sequence and they can form fiber structures through self-assembly with effects such as pH change, ionic strength and presence of oppositely charged molecules [14]. In aqueous environment, these fibers can form 3D networks at the micro level and form gels that encapsulate water at the macro level. By placing biologically active amino acid sequences in the peptide amphiphile structure, the functionally significant protein structure of the extracellular matrix can be mimicked, and a suitable environment can be provided for the cells to proliferate or differentiate as desired. In this study, we have used glycosaminoglycan-mimetic functional groups that bind to and increase the local concentration of growth factors released from resident cells [25]. The chemical structures of the peptide amphiphile molecules synthesized are shown in in Fig. 1. Oppositely charged peptide amphiphile molecules mixed at physiological pH form a nanofibrous network structure through electrostatic and hydrophobic interactions [30]. Liquid chromatography (LC) and mass spectroscopy (MS) results of the synthesized peptides revealed that the molecular weights of the peptides corresponded to the theoretically calculated values showing that the peptides were synthesized correctly (Supplementary Fig. S1). The single and dominant peak in the HPLC results indicated that the synthesized peptides were pure and the purity ratio was higher than 96% (Stable 1). In this study, four different hydrogels were formed by mixing positively charged K-PA, negatively charged E-PA and/or SO_3_-PA at different ratios. These combinations and the ratios used are presented in Supplementary Table S2.

Scanning electron microscopy was used to visualize the nanofiber networks forming the hydrogels. The nanofiber structure showed similar structural features to the natural extracellular matrix, such as matrix fibril diameter (10 nm to 300 µm) and porosity (0.1×10^−8^ to 1×10^−9^) (Fig. 1). In order to examine the secondary structures of the nanofibers, CD analysis was used. The characteristic peaks shown by the secondary structures in the CD spectrum are as shown in Fig. 1. At physiological pH, E-PA and SO_3_-PA are both negatively charged and showed a characteristic random coil spectrum individually. The positively charged K-PA also showed an irregular helix spectrum similar to the negatively charged peptides. When the positively and negatively charged peptides were mixed, the irregular helix signals disappeared and a characteristic β-sheet structure signal (maxima; 195 nm, minima; 220 nm) was observed, showing that the dominant secondary structure in the nanofibers structure is the β-sheet structure. Zeta potential measurements further validated the establishment of self-assembly process, as the mixing of two oppositely charged PA molecules decreased the stability of the individual solutions by 30 mV, indicating aggregations due to self-assembly at pH 7.4.

### Cellular viability, adhesion and spreading analysis

**Figure 2.**
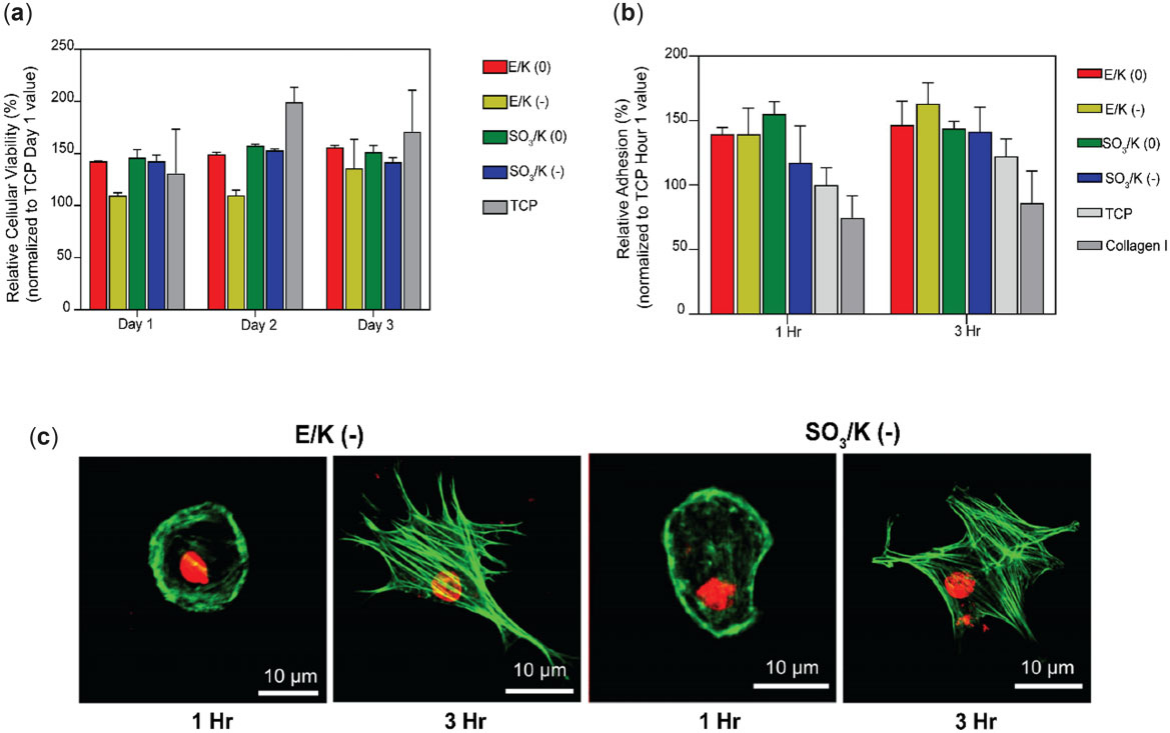
(**a**) Viability analysis of mMSCs cultured on indicated platforms for Days 1, 2 and 3. Results normalized to Day 1 TC*P* values. (**b**) Adhesion analysis of mMSCs seeded on indicated coatings at 1 and 3 h. Results normalized to Hour 1 TC*P* values. (**c**) Representative spreading of mMSCs on E/K (−) and SO_3_/K (−). Spreading of mMSCs showed by actin fiber staining (nuclei stained with to-PRO3, actin fibers stained with TRITC-conjugated phalloidin). Values represent mean±SEM, *n* = 3 (***P < 0.0001, **P < 0.01, *P < 0.05).

The initial responses of cells to peptide nanofibers were analysed with cell attachment, cell viability and cell spreading experiments. Cellular viability was examined on Days 1, 2 and 3. The number of cells seeded on nanofiber coatings was at comparable levels with the number of cells seeded on bare tissue culture plate (TCP) on Days 1, 2 and 3 suggesting that the peptide nanofibrous networks are biocompatible (Fig. 2a). Cells were able to adhere to the peptide nanofibers and changed their morphology following their seeding, as shown by actin staining (Fig. 2c). The number of attached cells to the peptide nanofiber scaffolds was more than the number of cells attached on the TCP and collagen I coated surface at the first and third hours. Although there was no significant difference between the nanofiber scaffolds in terms of cellular attachment, the rate of cell attachment was observed to increase after 3 h of culture with respect to 1 h of culture. This result shows that nanofiber scaffolds do not inhibit cell attachment and create a suitable environment for cells to adhere (Fig. 2b).

### Differentiation analysis of mouse mesenchymal cells cultured on peptide nanofiber coatings

Mesenchymal stem cells can differentiate into chondrocytes in the presence of growth factors such as TGF-β and BMP in *in vitro* conditions. In this study, we first investigated the chondrogenic differentiation potential of the mesenchymal stem cells in the presence of varying growth factor concentrations when cultured on extracellular matrix-mimetic coatings. Four different culture media were used of which three of them included varying amounts of growth factors (Supplementary Table S3).

To examine the long-term responses of the mesenchymal stem cells, cells were cultured on the peptide nanofiber coatings for longer durations. During embryonic development, stem cells increase their cell–cell interactions and form clumps/aggregates to induce cartilage differentiation. This kind of aggregate formation is accepted as an indicator of differentiation for mesenchymal stem cells. In order to investigate whether there were similar aggregate formations, cellular morphologies were monitored in different culture media for 14 days (Supplementary Fig. S4). Light microscopy images revealed that the mesenchymal stem cells cultured on peptide nanofiber coatings increased their cell–cell interactions and cells showed a tendency to form aggregates. It is important to note that the cells cultured on peptide nanofibers but with the medium with reduced growth factor concentration also showed aggregate formation in varying numbers and sizes. This result may be caused by the effect of the nanofibers on enhancing growth factor deposition and presentation. When cells cultured on different peptide nanofiber coatings were compared, coatings bearing more negative charges showed have a prominent effect on pre-cartilage aggregate formation. The cells cultured on SO_3_/K (−) showed round morphology and tended to clump more, similarly to cells cultured on E/K (−), in chondrogenic media. Moreover, the clusters observed on the SO_3_/K (−) were larger and distinct than the clusters observed on the SO_3_/K (0) (Supplementary Fig. S4). This result showed that even if there are similar functional groups on coatings, overall charge ratio might have a strong effect on cluster formation and chondrogenic differentiation.

**Figure 3.**
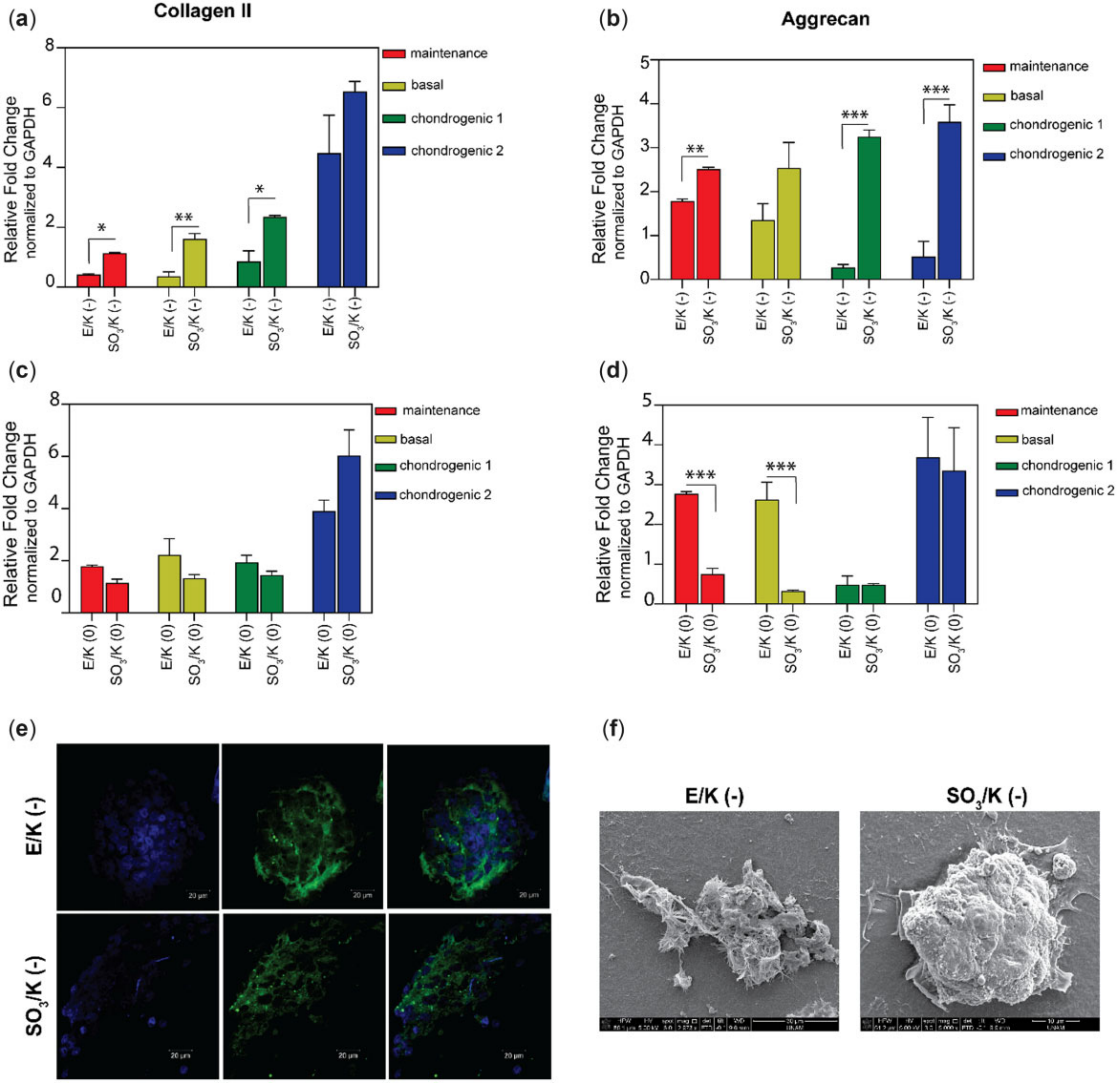
(**a**, **c**) Collagen II (**b**, **d**) aggrecan expression of mMSCs on indicated coatings on Day 7 in maintenance, basal, chondrogenic medium 1 and 2. The expression level of each gene was normalized against uncoated tissue culture plate samples (TCP) and GAPDH was used as the internal control. Values represent mean±SEM, *n* = 3 (***P < 0.0001, **P < 0.01, *P < 0.05). (**e**) mMSCs cultured on E/K (−) and SO3/K (−) coatings express cartilage specific collagen II proteins on Day 7. Collagen II were labeled with Cy3 secondary antibody and cell nuclei were labeled with DAPI^®^-3. (**f**) SEM images of aggregates formed by mMSCs cultured on E/K (−) and SO3/K (−) coatings on Day 7. Values represent mean±SEM, *n* = 3 (***P < 0.0001, **P < 0.01, *P < 0.05).

The cells entering the chondrogenic differentiation pathway increase glycosaminoglycan synthesis and produce marker proteins such as collagen II and aggrecan. Extracellular matrix depositions were first analysed by using scanning electron microscopy which revealed that cells formed aggregates and deposited extracellular matrix on nanofibrous scaffolds (Fig. 3d). Then, aggrecan and collagen II expression in the extracellular matrix were investigated by immunocytochemistry, where it was observed that the cells cultured on both SO_3_/K (−) and E/K (−) showed higher Collagen II and Aggrecan deposition in their extracellular matrix compared to cells cultured on tissue culture plates (Fig. 3c and Supplementary Fig. S5).

Next, the expression levels of cartilage marker genes were examined through qRT–PCR analyses during 14 days of culture (Fig. 3 and Supplementary Fig. S6). On Day 7, both Collagen II and Aggrecan expressions were significantly higher in cells cultured on SO_3_/K (−) nanofibers than cells cultured on E/K (−) nanofibers in all medium compositions (Fig. 3a and b). However, there was no significant difference in Collagen II expression levels between the cells cultured on E/K (0) and SO_3_/K (0) nanofibers. Yet, cells cultured on E/K (0) nanofibers showed higher Aggrecan gene expression than cells cultured on SO_3_/K (0) nanofibers.

When Collagen II expression levels were analysed on Day 14 (Supplementary Fig. S5), the reverse of the pattern observed on Day 7 was observed. Cells cultured on E/K (−) nanofibers showed higher Collagen II and Aggrecan gene expressions than cells cultured on SO_3_/K (−). A similar situation was also observed for cells cultured on E/K (0) and SO_3_/K (0) nanofibers. These results show that the cells cultured on SO_3_/K (−) nanofibers differentiated into cartilage cells faster than cells cultured on E/K (−) nanofibers. Moreover, it is important to note that even cells cultured in medium with no growth factor could enter the chondrogenic differentiation pathway and synthesize cartilage-specific marker proteins when cultured on bioactive peptide nanofibers. This result showed that these bioactive peptide nanofibers can enhance the chondrogenic differentiation of cells even in the absence of any supportive growth factors. Moreover, mesenchymal stem cells committed to chondrogenic differentiation pathway on all combinations of peptide amphiphile nanofibers that some of them have no sulfated groups. This result showed that the sulfated groups might have an accelerator role and that peptide amphiphile nanofibers with sulfated groups induced cartilage marker gene expression more than groups with no sulfated groups on Day 7 [31].

The earlier cartilage differentiation of cells and higher marker protein expressions in the groups with negative charges, especially the sulfated group, are consistent with other cartilage differentiation studies in the literature focusing on charge effect. Negative charges are extremely crucial in the development and maintenance of cartilage tissue; first, the negative charges of sulfated hydrogels provide chemical cues to embedded cells that help generate and maintain a chondrogenic phenotype. Secondly, the negative charges of sulfated hydrogels allow for the prolonged release of positively charged growth factors via electrostatic interaction. These diffusible signaling proteins promote cell proliferation, differentiation and survival, as well as modify and regulate inflammation and tissue repair [31–33].

### Differentiation analysis of mouse mesenchymal cells cultured in 3D peptide nanofiber hydrogels

**Figure 4.**
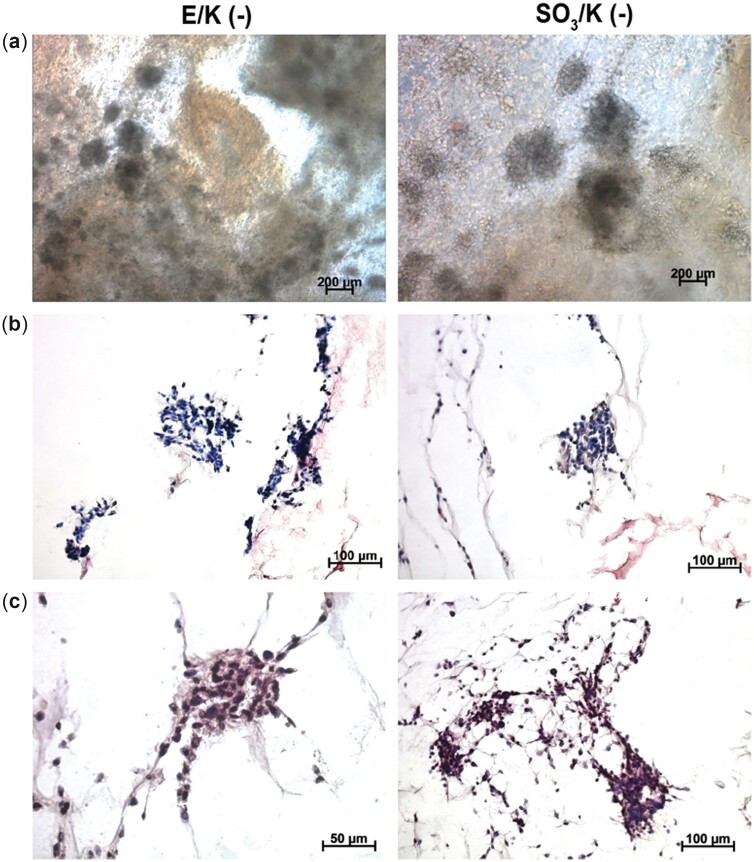
(**a**) Light microscope images of mMSCs cultured in E/K(−) and SO_3_(−) hydrogels on Day 14. (**b**) Hematoxylin and eosin stainings showing aggregate formation by mMSCs cultured in 3D E/K(−) and SO_3_(−) hydrogels on Day 14. (**c**) Safranin-O stainings showing glycosaminoglycan depositions by mMSCs cultured in E/K(−) and SO_3_(−) hydrogels on Day 14. Values represent mean±SEM, *n* = 3 (***P < 0.0001, **P < 0.01, *P < 0.05).

**Figure 5.**
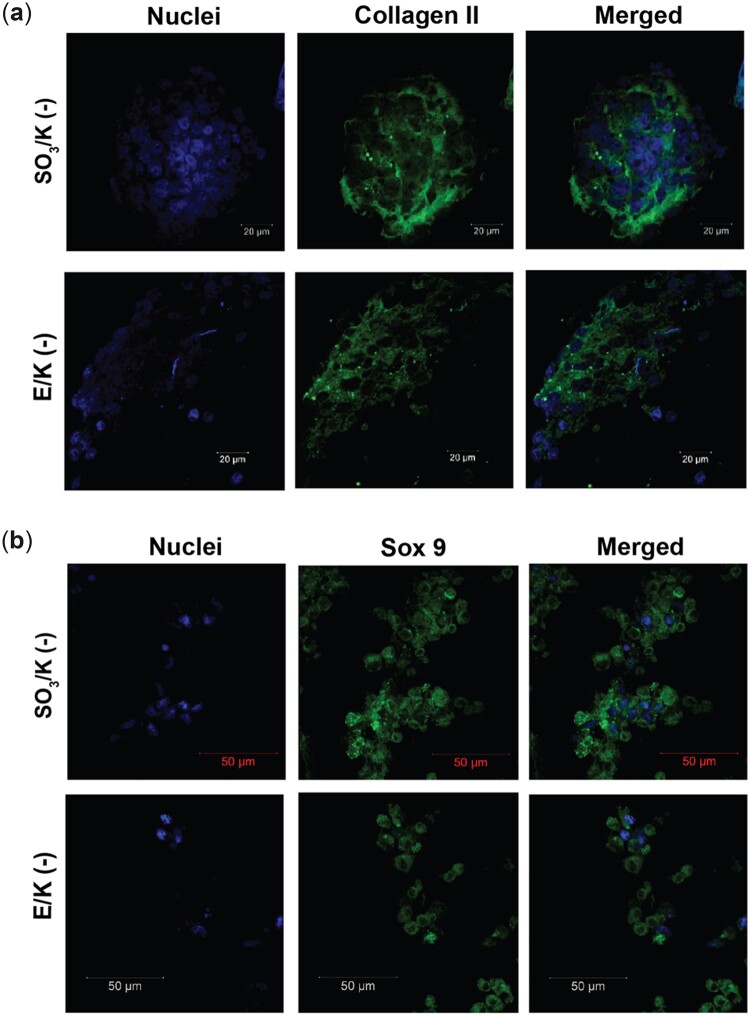
Immunofluorescence collagen II (**a**) and SOX 9 (**b**) staining on sections of mMSC cultured in 3D E/K(−) and SO_3_(−) hydrogels on Day 14. Collagen II and SOX 9 were labeled with Cy3 secondary antibody and cell nuclei were labeled with DAPI^®^-3. Values represent mean±SEM, *n* = 10 (***P < 0.0001, **P < 0.01, *P < 0.05).

3D culture systems are important to mimic the extracellular niche to enable higher rates of cellular interactions and growth factor depositions, directed differentiation of cells and keeping cells in their differentiated state. For analysing the effect of 3D cultures in peptide nanofiber hydrogels, mesenchymal stem cells were incubated in all gel formulations. All mesenchymal stem cells were cultured in maintenance medium with no exogenous growth factor to mimic the possible clinical applications of these hydrogels as addition of exogenous growth factors is undesirable due to their carcinogenic properties. Hydrogels were prepared by using 10 nm peptide amphiphile solutions as mentioned above and cell solutions were added into the gels during gel preparation to attain a homogenous culture and to keep gels intact as shown in Supplementary Fig. S7. Using oscillatory rheology, the mechanical characteristics of nanofibrous hydrogels were investigated. To assess the viscoelastic behaviors of PA 3D hydrogels, a time sweep test was undertaken by recording the storage (G′) and loss (G′′) moduli of the hydrogels for 1 h at constant shear strain and angular frequency. Storage moduli were greater than loss moduli for E/K (−) and SO_3_/K (−) hydrogels produced in water and in HEPES buffer. This result indicated that the hydrogels exhibited elastic solid behavior (Supplementary Fig. S7). Hydrogels were cultured for 14 days in static condition. On Day 14, the cells were observed to clump together and form distinct clusters (Fig. 4). Cells also synthesized cartilage-like extracellular matrix evidenced by glycosaminoglycan deposition and expression of cartilage markers such as Collagen II and Sox 9 (Fig. 5; Supplementary Figs. S8 and S9). This result showed that similar to 2D culturing on peptide nanofibers, 3D hydrogel culture was an inducing environment for the differentiation of mesenchymal stem cells into chondrocytes. Moreover, the 3D culturing enabled cells to form aggregates and synthesize cartilage-specific proteins without the use of any growth factor. However, in parallel with the 2D results, gel formulations having extra negative charges (E/K (−) and SO_3_/K (−)) showed more distinctive aggregates than neutral groups. Glycosaminoglycan deposition was also analysed and the samples treated with chondroitinase showed faint staining (Supplementary Fig. S10). Chondroitinase treatment analysis confirmed that the Safranin-O staining was positive, indicating that glycosaminoglycan synthesis occurs in the cells’ extracellular matrix.

## Conclusion

In this study, we cultured mesenchymal stem cells in 2D and 3D platforms that provide a bioactive environment for stem cells to differentiate into cartilage cells. It is proposed that negatively charged molecules similar to glycosaminoglycans play a critical role for mesenchymal stem cell differentiation similar to the natural stem cell environment. During embryonic chondrogenesis, stem cells form aggregates through induction by small molecules, and change their morphology to increase cell–cell interactions. In this study, we tried to mimic such an environment for mesenchymal stem cells to induce their chondrogenic differentiation. Peptide amphiphile nanofibers are able to present specific functional groups in a dense manner and encapsulate large amounts of water forming hydrogels. Our study showed that peptide nanofibers decorated with small functional groups like sulfate, hydroxyl and carboxylate could easily induce mesenchymal stem cell chondrogenic differentiation without the need for growth factors or small inducer molecules like dexamethasone, insulin and transferrin. Having similar results for 2D and 3D culturing pave the way for the use of these platforms for developing different types of organoids and lab-on-a-chip applications. For degenerative cartilage diseases, it is quite significant to attain satisfactory cell populations to build new tissue at the degenerated site. These platforms could be useful both for engaging mesenchymal stem cells to the site of interest and inducing their differentiation to form new functional tissues during treatment.

## Supplementary data

Supplementary data are available at *Regenerative Biomaterials* online.

*Conflicts of interest statement*. The authors declare that they have no known competing financial interests or personal relationships that could have appeared to influence the work reported in this paper.

## Supplementary Material

rbac084_Supplementary_Data
