# Correction to: Sulfated GAG mimetic peptide nanofibers enhance chondrogenic differentiation of mesenchymal stem cells in 3D *in vitro* models

**DOI:** 10.1093/rb/rbaf045

**Published:** 2025-05-22

This is a correction to: Seher Yaylaci, Mustafa O Guler, Ayse B Tekinay, Sulfated GAG mimetic peptide nanofibers enhance chondrogenic differentiation of mesenchymal stem cells in 3D *in vitro* models, *Regenerative Biomaterials*, Volume 10, 2023, rbac084, https://doi.org/10.1093/rb/rbac084

During an internal review, the authors identified that the circular dichroism (CD) spectra and scanning electron microscopy (SEM) images in Figure 1(a) and Figure 1(d) were inadvertently reused from a previously published study (*DOI: 10.1021/bm301538k*). This error occurred due to a file mismanagement issue during the manuscript preparation phase, where incorrect datasets were incorporated into the final submission.

The purpose of including the CD and SEM data in Figure 1 was to provide supporting evidence for the structural and morphological characteristics of the peptide amphiphile (PA) nanofibers used in this study. While the PA nanofibers in this work are chemically identical to those in the previously published study and exhibit consistent β-sheet structures and fibrillar morphology, the authors acknowledge that the data presented in the original submission should have been derived exclusively from the correct dataset associated with this study.

The corrected Figure 1, generated from the appropriate dataset originally obtained for this study, is shown below. No new measurements were performed; rather, the correct data from this study were selected and used to construct the revised figures.

This correction does not affect the primary results or conclusions of the study, as the core findings—focused on the chondrogenic differentiation of mesenchymal stem cells in 3D environments—are based on entirely new and independent datasets. The revised figures accurately reflect the experimental data generated in this study.

Corrected Figure 1:

**Figure:**
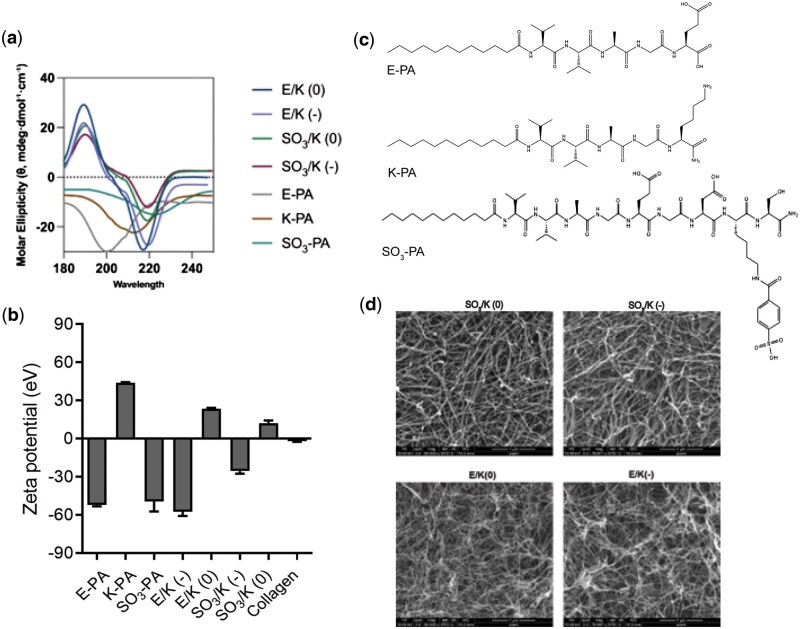


These details have been corrected only in this correction notice to preserve the published version of record.

