# Supplementary material for: Sulfated GAG mimetic peptide nanofibers enhance chondrogenic differentiation of mesenchymal stem cells in 3D in vitro models
Source: Regen Biomater. 2022 Nov 7;10:rbac084. doi: 10.1093/rb/rbac084 (PMC9847523; doi:10.1093/rb/rbac084)
Supplement: rbac084_Supplementary_Data [file rbac084_supplementary_data.docx]

SUPPORTING INFORMATION FOR

**Sulfated GAG Mimetic Peptide Nanofibers Enhance Chondrogenic Differentiation of Mesenchymal Stem Cells in Three-Dimensional In Vitro Models**


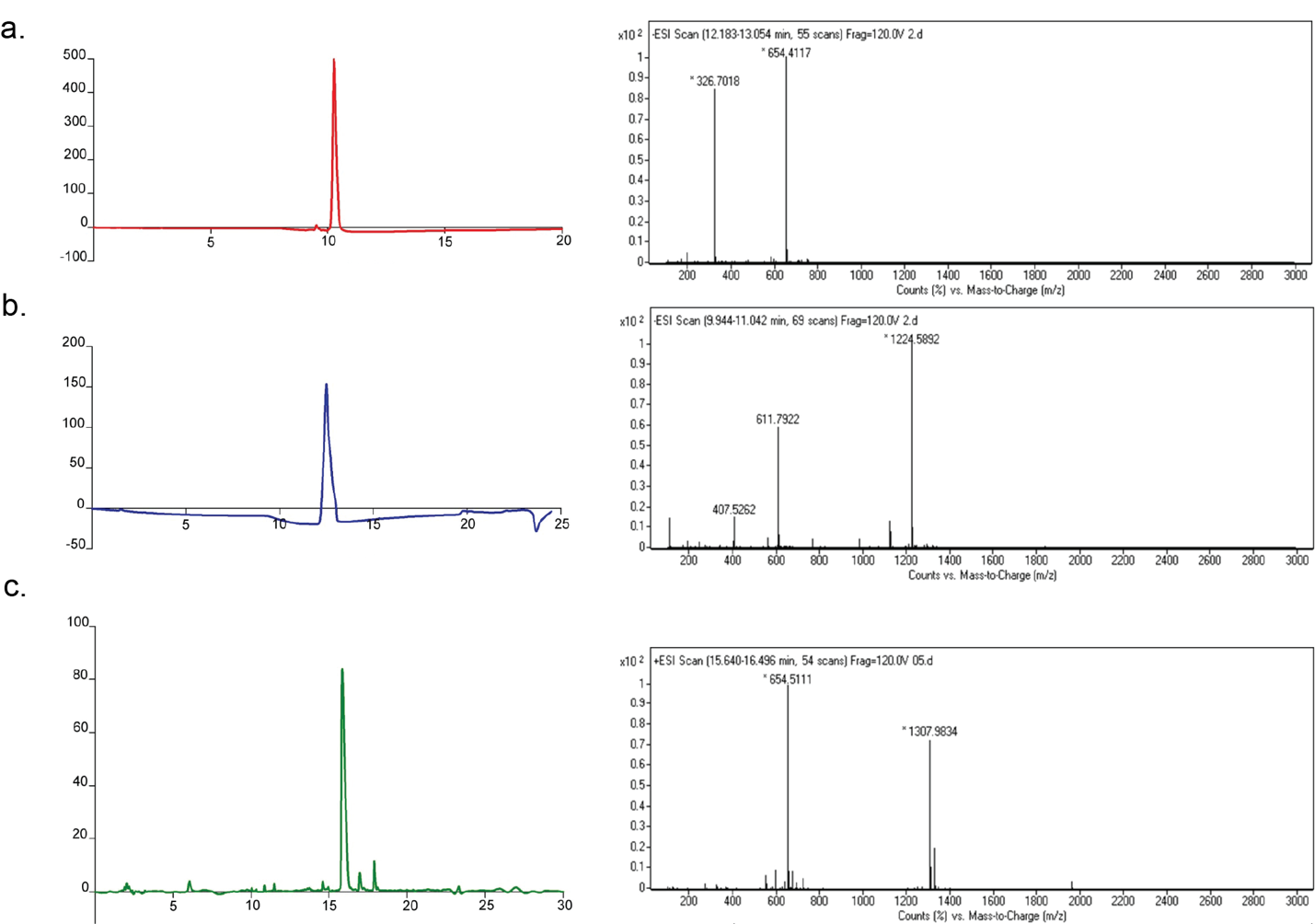


**FIGURE S 1.** Reverse-HPLC chromatogram and Liquid chromatography-mass spectrometry (LC-MS) analyses of E-PA (a), SO_3_-PA (b) and K-PA(c).


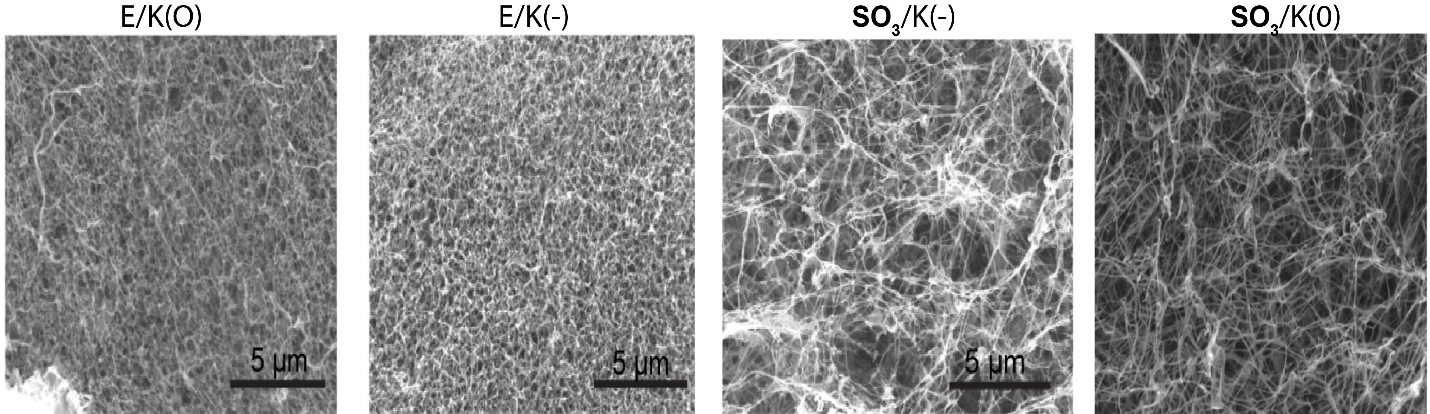


**FIGURE S 2** Scanning electron microscope micrograph of indicated nanofibers.

**Table S 1** Sequences, molecular weights and net charges and HPLC Analysis of the peptide amphiphile molecules at pH 7

| **PA**  **molecule** | **PA sequence** | **Molecular weight**  **(g/mol)** | **Net Charge (pH7)** | **Retention Time (min)** | **Purity (area %)** |
| --- | --- | --- | --- | --- | --- |
| SO_3_-PA | C12-VVAGEGD(K-pbs)S | 1226 | -3 | 10.8 | 97.1 |
| E-PA | C12-VVAGE | 655,82 | -2 | 12.7 | 96.4 |
| K-PA | C12-VVAGK | 654 | +1 | 15.4 | 96.8 |

**Table S 2** Composition and net loads of the nanofiber scaffolds

| **PA combination** | **Volume ratio** | **Net Charge (pH7)** |
| --- | --- | --- |
| SO_3_/K(0); SO_3_-PA/K-PA | 1:3 | Neutral |
| SO_3_/K(-); SO_3_-PA/K-PA | 1:2 | Negative |
| E/K(0); E-PA/K-PA | 1:1 | Neutral |
| E/K(-); E-PA/K-PA | 2:1 | Negative |

**Table S 3** Different media and their contents when culturing msenchymal stem cells on peptide nanofibers

| **Maintenance medium** | 10% FBS + DMEM |
| --- | --- |
| **Basal medium** | 10^-7^M Dexamethazone, 1 µM, Transferrin, 6 μg/mL insulin, 3x10^-5^ M Sodium Selenite, 0.5mg/mL BSA+ DMEM |
| **Chondrogenic medium 1** | 10^-7^M Dexamethazone, 1 µM, Transferin, 6 μg/mL insulin, 3x10^-5^ M Sodium Selenite, 0.5 mg/mL BSA+ DMEM + **2 ng/mL TGF beta-1** |
| **Chondrogenic medium 2** | 10^-7^M Deksamethazone, 1 µM, Transferin, 6 μg/mL Insulin, 3x10^-5^ M Sodium Selenite, 0.5 mg/mL BSA+ DMEM + **10 ng/mL TGF beta-1** |

***
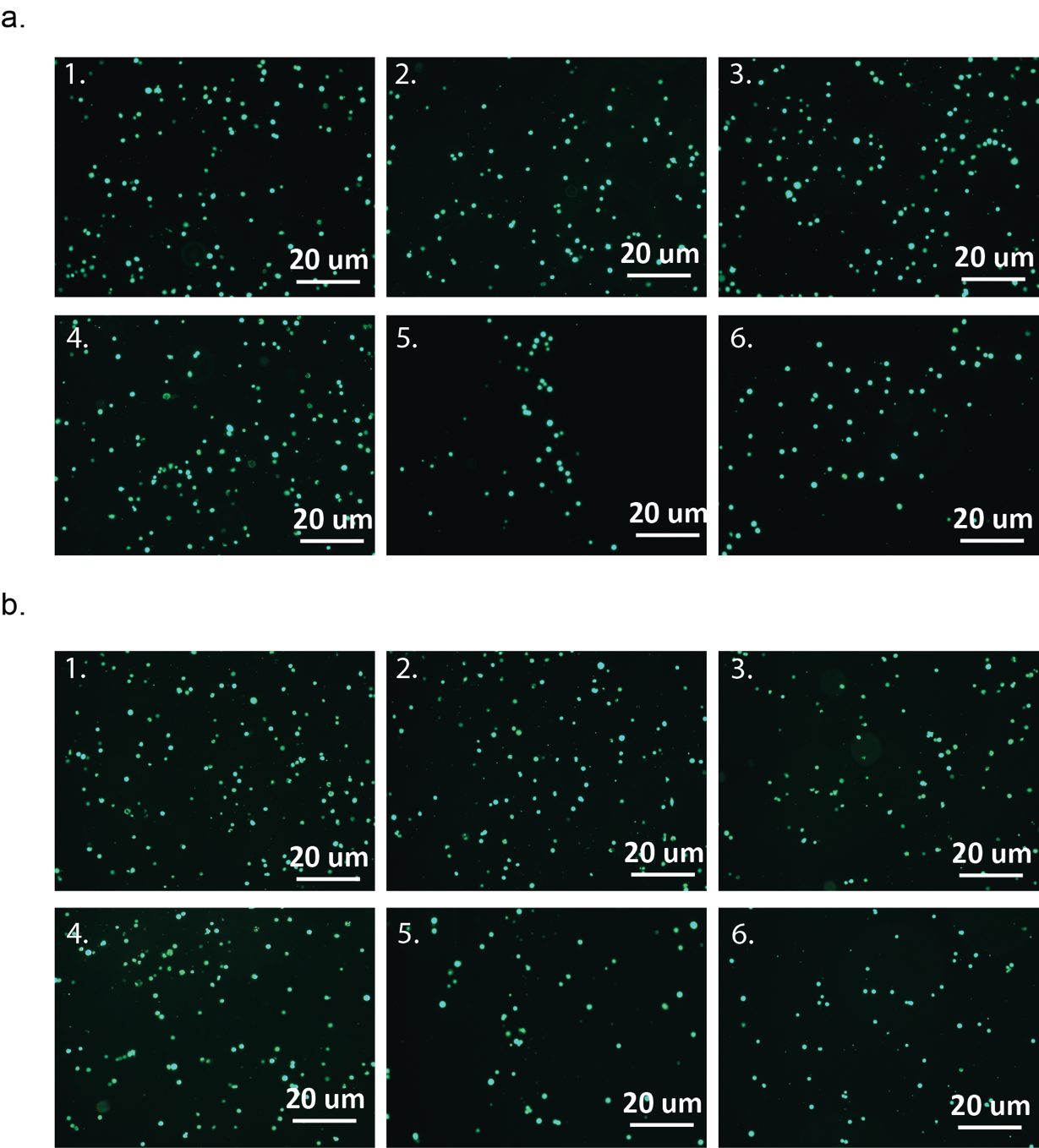
***

**FIGURE S 3** Representative images of adhered live mouse mesenchmal stem cells at (a) 1 h and (b) 3 h of culture on various coatings (1; E/K (0), 2;E/K (-), 3;SO_3_/K (0), 4;SO_3_/K (-), 5;TCP and 6; Collagen I. Live cells stained with Calcein AM (green).


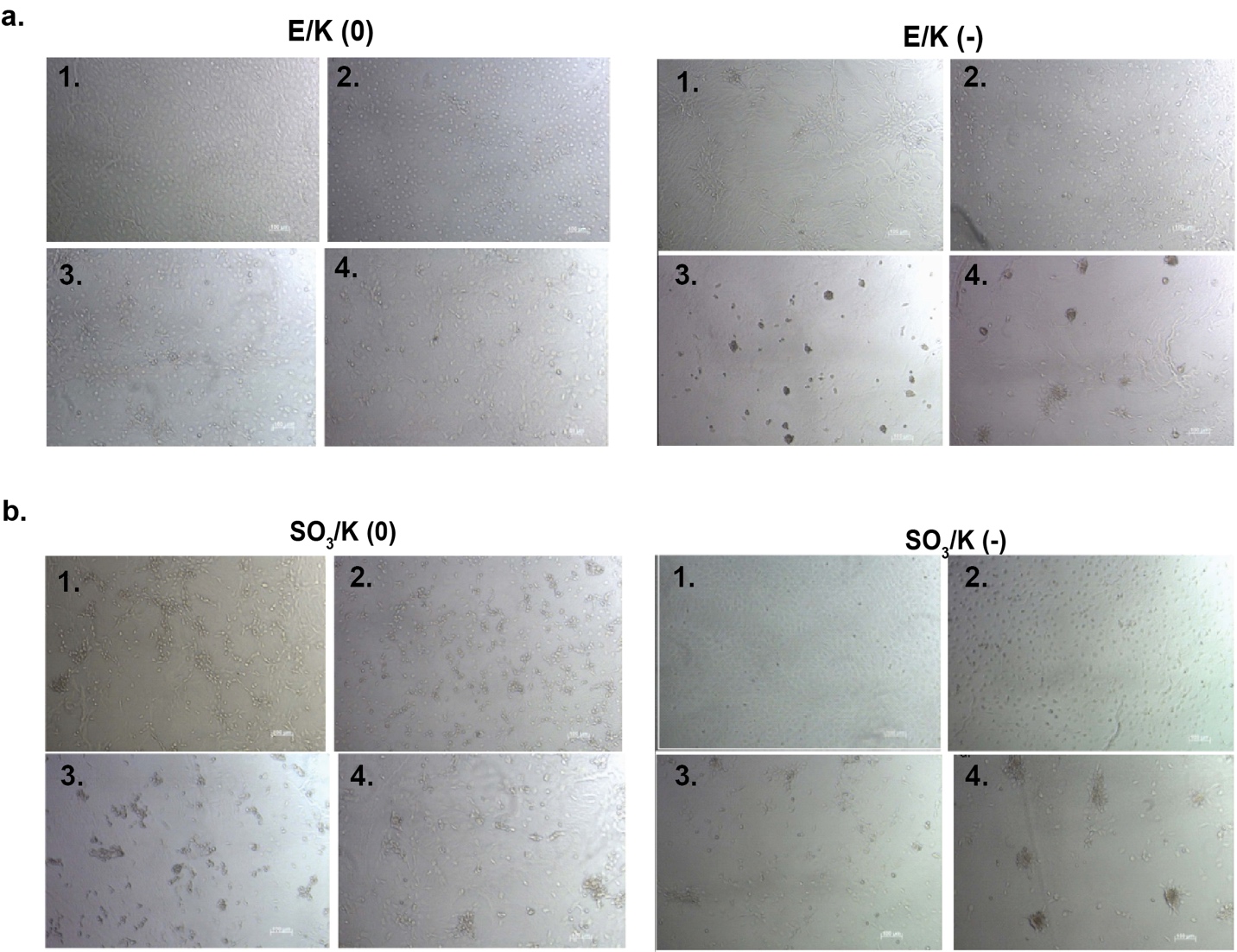


**FIGURE S 4** Light microscope images of mouse mesenchymal stem cells cultured on (a) E/K (0), E/K (-) and (b) SO_3_/K(0), SO_3_/K(-) coatings in 1; maintenance medium, 2; basal medium, 3; chondrogenic medium 1 and 4; chondrogenic medium at day 7.


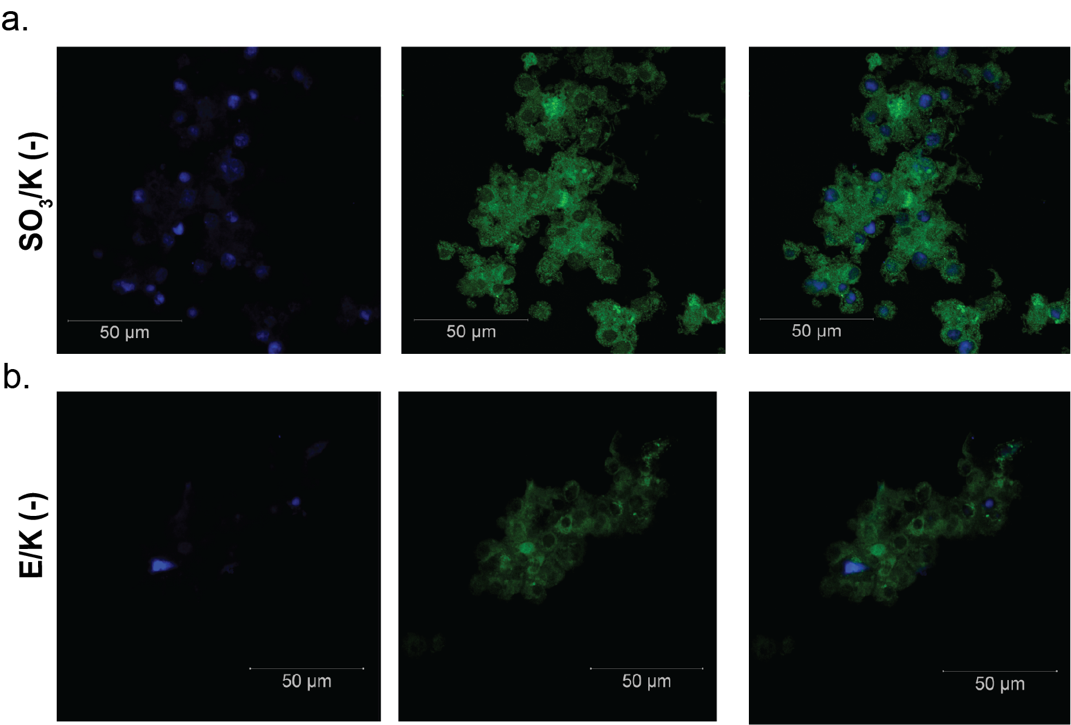


**FIGURE S 5** Mouse mesenchymal stem cells cultured on E/K (-) and SO_3_/K (-) coatings express cartilage specific Aggrecan proteins on day 7. Collagen II were labeled with Cy3 secondary antibody (green) and cell nuclei were labeled with DAPI®-3 (blue).

**
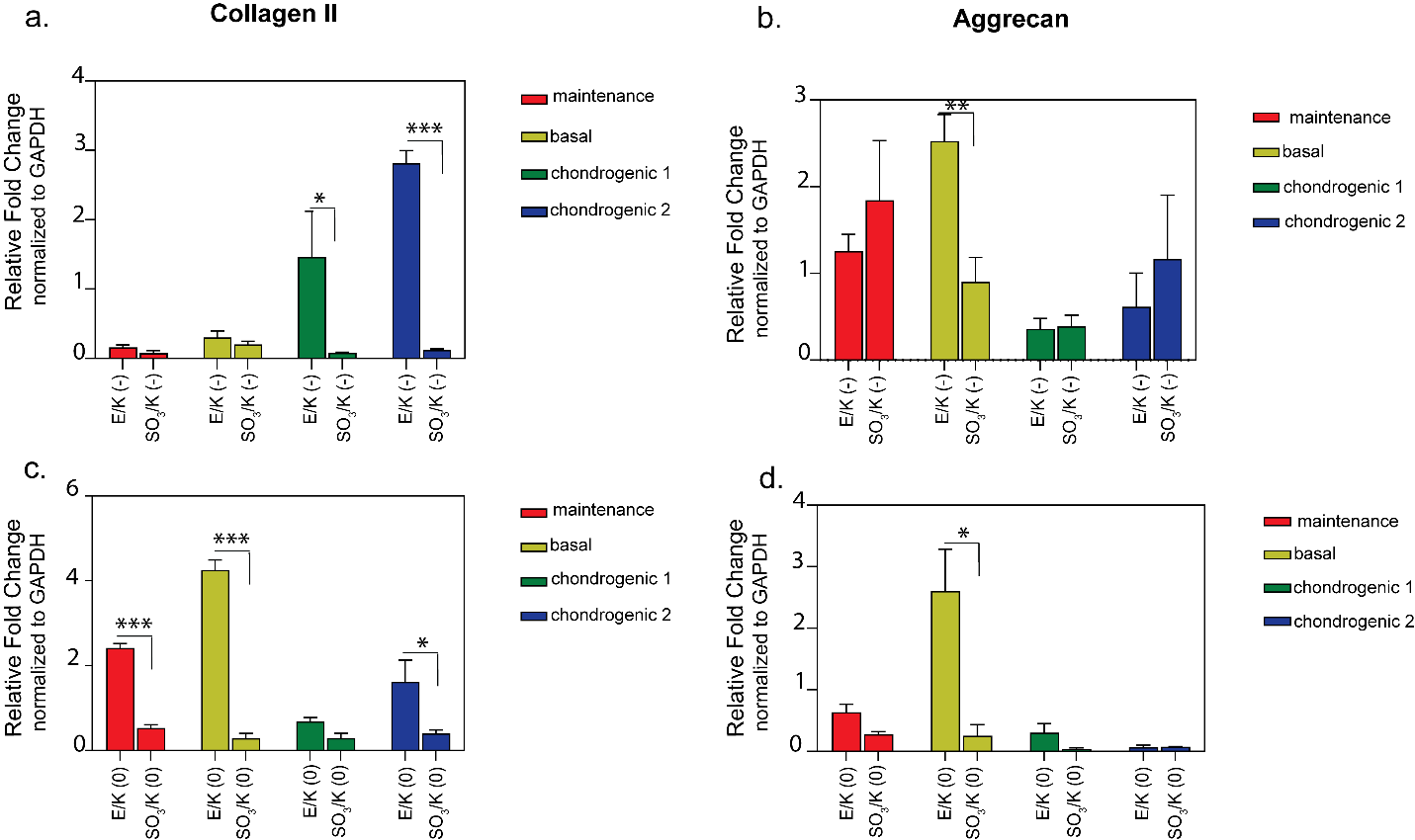
**

**FIGURE S 6** (a, c) Collagen II (b, d) Aggrecan expression of mouse mesenchymal stem cells on indicated coatings (a, b) on day 7 and (c, d) on day 14 in maintenance, basal, chondrogenic medium 1 and 2. The expression level of each gene was normalized against uncoated tissue culture plate samples (TCP) and GAPDH was used as the internal control. Values represent mean ± SEM, n = 3 (***p < 0.0001, **p < 0.01, *p < 0.05).


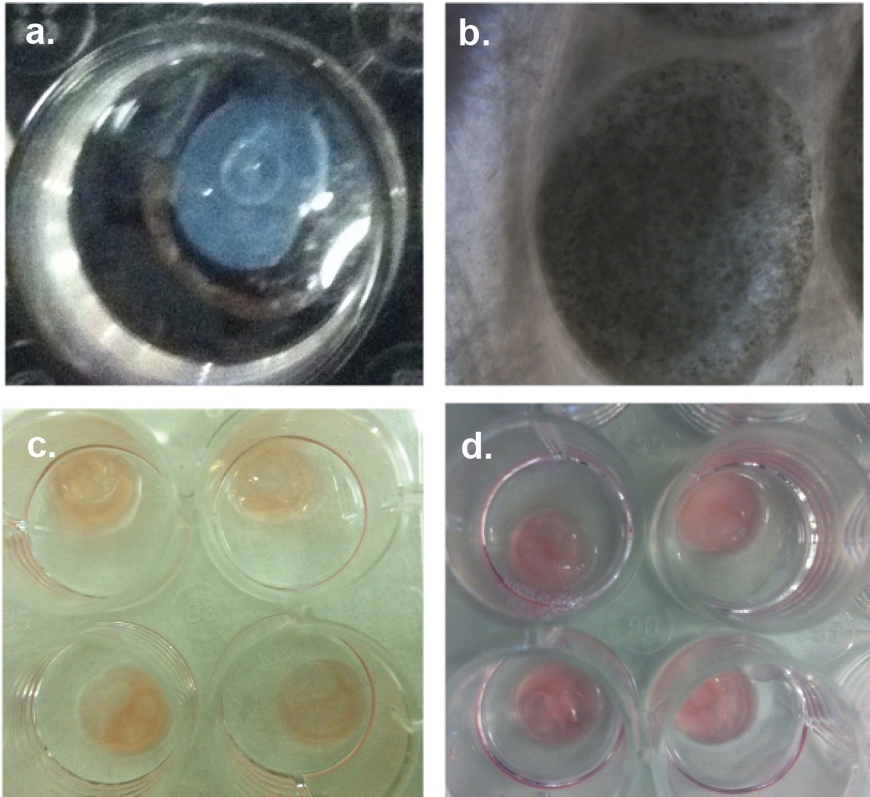

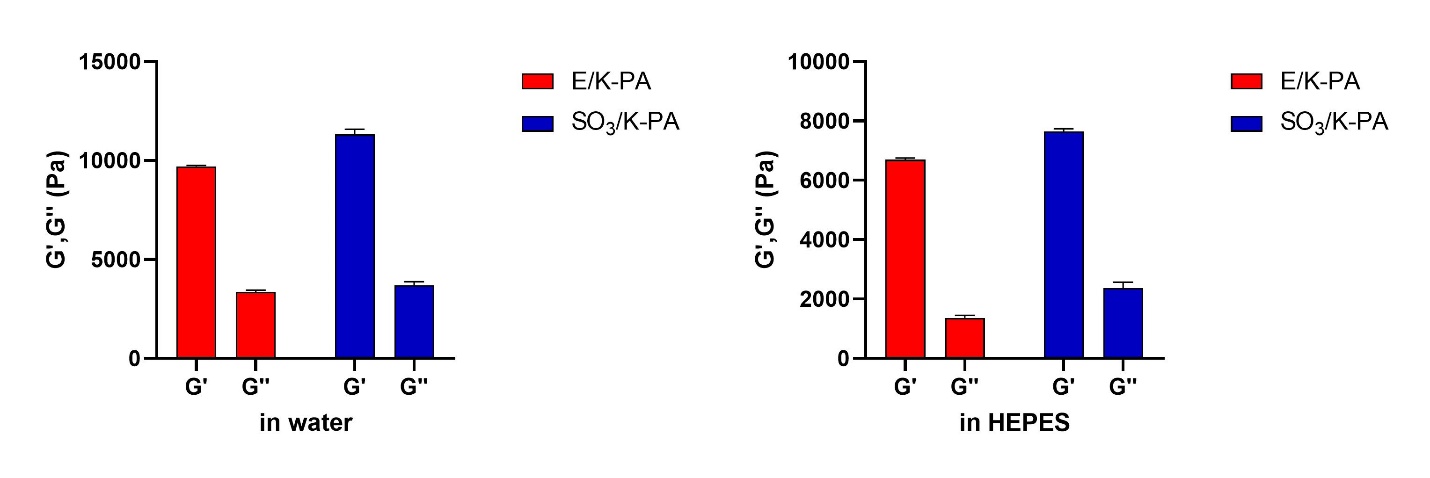


e.

f.

**FIGURE S 7** (a) 3D culture formation by mixing peptide amphiphiles in a well of 24-well plate. (b) Light microscope images of seeded mouse mesenchymal stem cells in 3-dimensional hydrogels. Images of 3-dimensional hydrogel on day 3 (c) and on day 7 (d) Storage and loss modulus of E/K(-) and SO_3_(-) hydrogels in water (e) and HEPES buffer (f) at pH 7.


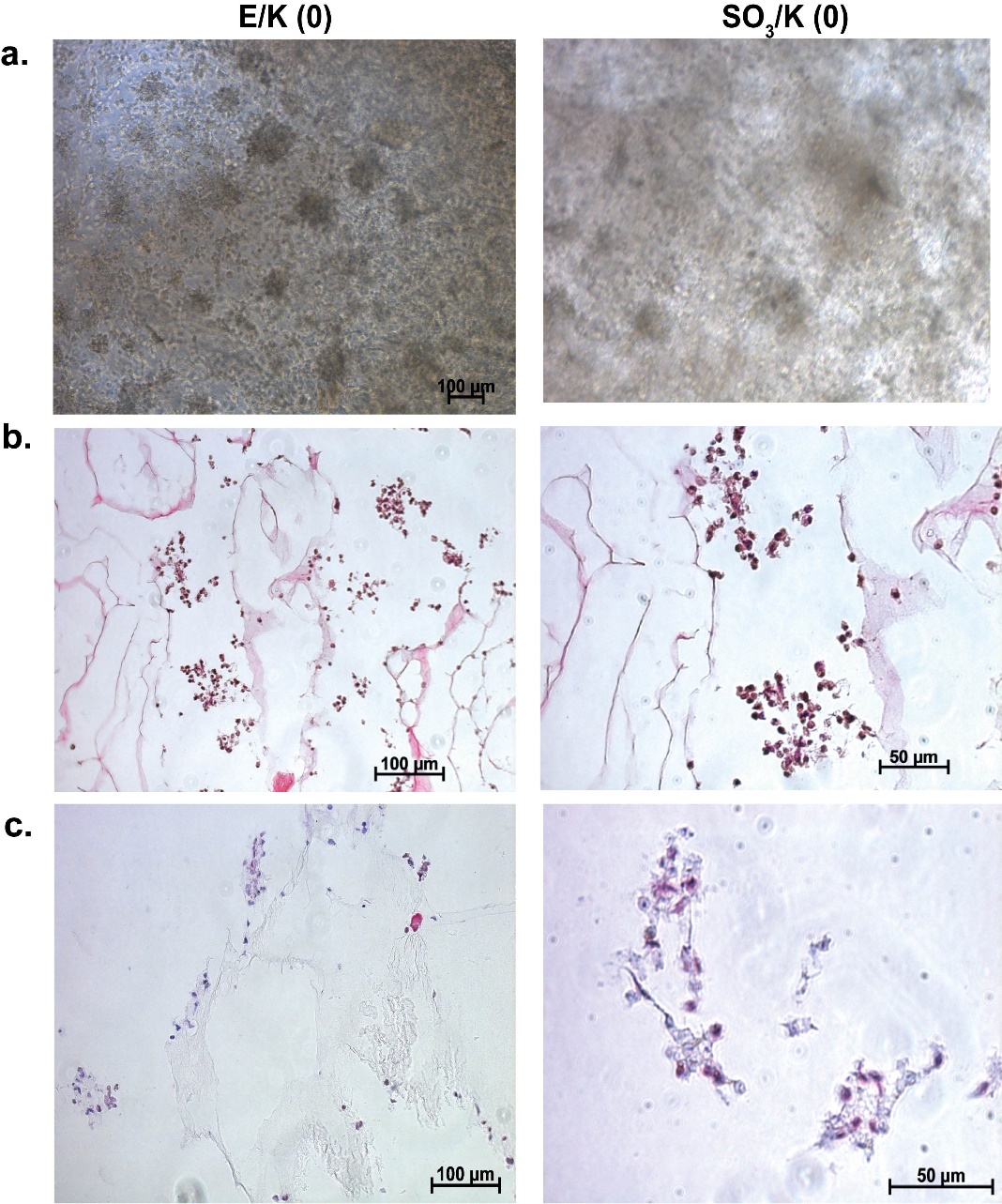


**FIGURE S 8** (a) Light microscope images of mMSCs cultured in E/K(0) and SO_3_(0) hydrogels on day 14. (b) Hematoxylin and eosin stainings showing aggregate formation by mMSCs cultured in 3D E/K(0) and SO_3_(0) hydrogels on day 14. (c) Safranin-O stainings showing glycosaminoglycan depositions by mMSCs cultured in E/K(0) and SO_3_(0) hydrogels on day 14.

*
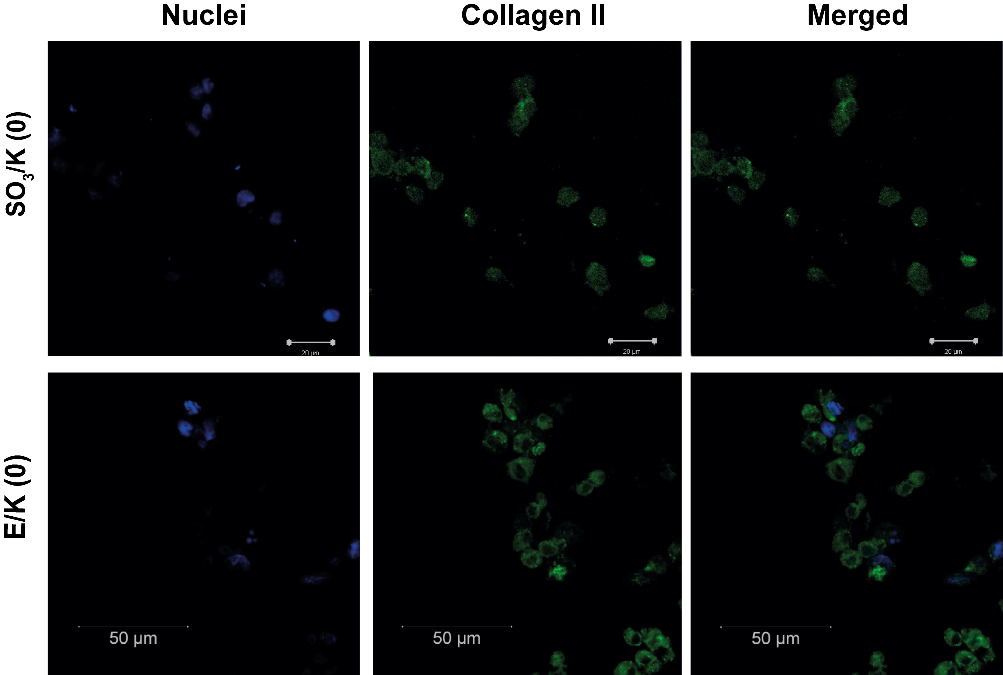
*

**FIGURE S 9** Immunofluorescence collagen II staining on sections of mMSC cultured in 3D E/K(0) and SO_3_(0) hydrogels on day 14. Collagen II were labeled with Cy3 secondary antibody (green) and cell nuclei were labeled with DAPI®-3 (blue).


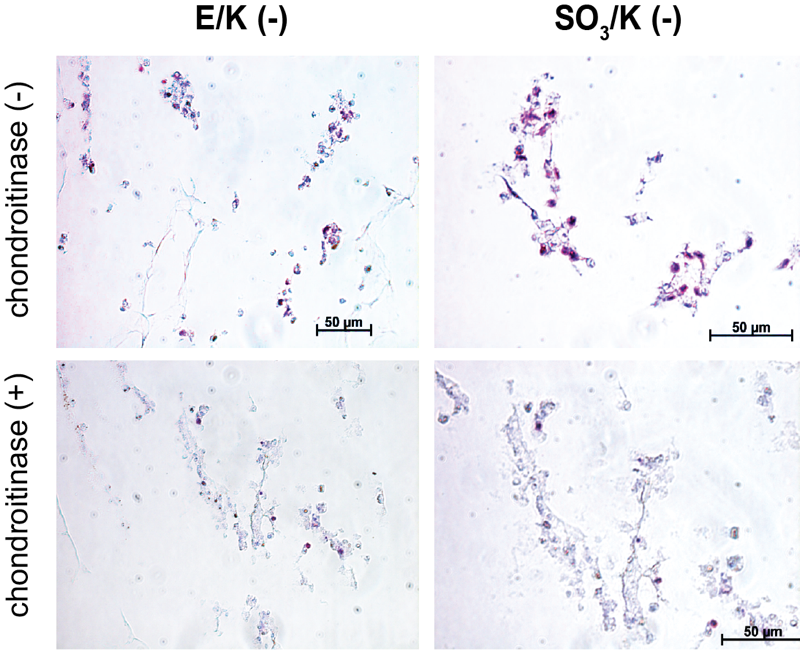


**FIGURE S 10** Safranin-O stainings of mouse mesenchymal stem cells cultured in 3-dimensional E/K(-) and SO_3_(-) hydrogels on day 14 incubated with and without chondroitinase before Safranin-O staining.
